# Establishment of an inflammatory cytokine-based predictive model for the onset of prolonged postoperative ileus after radical gastrectomy: a prospective cohort study

**DOI:** 10.3389/fimmu.2025.1552944

**Published:** 2025-03-24

**Authors:** Chao Sui, BeiBei Wang, Yu Zhao, YunTian Guo, JinXin Zhu, Feng Yu, XiaoDong Zhou, XueFeng Bu, Jie Zhang

**Affiliations:** ^1^ Department of General Surgery, Jiangsu University Affiliated People’s Hospital, Zhenjiang, China; ^2^ Department of General Surgery, Zhenjiang First People’s Hospital, Zhenjiang, China

**Keywords:** PPOI, IL-6, IL-10, TNF-α, nomogram

## Abstract

**Background:**

Prolonged postoperative ileus (PPOI) is a common postoperative abdominal complication and is strongly associated with the inflammatory response. However, there is a lack of effective means to predict PPOI in patients with gastric cancer.

**Methods:**

222 patients underwent radical gastrectomy at our center were enrolled and divided into the training group and validation cohort. Receiver operating characteristic (ROC) curve analysis and univariate and multivariable logistic regression models were performed to help filter variables for inclusion in the predictive model. And then a nomogram for PPOI was established. The area under the ROC curve (AUC) was calculated to assess the prediction accuracy. Diagnostic calibration curves were used to assess the goodness-of-fit of the nomogram. Decision Curve Analysis (DCA) was applied to evaluate its clinical utility.

**Results:**

Significant increase of IL-6, IL-10, TNF-α, and CRP on the first postoperative day were found in PPOI patients after surgery. Univariate and multivariate analysis demonstrated that age ≥ 65, IL-6, and IL-10 were independent predictive factors for PPOI. We subsequently developed a prediction nomogram of PPOI which included age, IL-6, IL-10, and TNF-α. Further verification by the training and validation groups demonstrated the good predictive efficacy of our model, as well as favorable clinical benefits.

**Conclusions:**

We developed a novel and easy-to-use prediction nomogram for gastric cancer, which was primarily based on the postoperative level of inflammatory mediators. This model provided further clarification of the exact relationship between inflammatory factors and the occurrence of PPOI, and help us clinically identify the high-risk groups of PPOI for the purpose of early intervention.

## Introduction

Postoperative ileus (POI), triggered by intraoperative gut manipulation and surgical trauma, is a common complication following radical gastrectomy (1). Generally, it gets relieved within three days, but may last for a longer duration, in which case it is known as prolonged postoperative ileus (PPOI), with an incidence of 3-32% (2, 3). During this phase, patients often suffer from vomiting, abdominal distension, and delayed defecation, which caused prolonged hospitalization time and increased expenses. It has been quite a burden on both inpatients and healthcare resources (4).

Currently, interventions such as Enhanced Recovery after Surgery (ERAS) program have been widely employed in radical gastrectomy. Emphasis on early intestinal rehabilitation and PPOI prevention is an important element of clinical practice guidelines (5, 6). Thus, identifying patients at high risk for PPOI is essential in order to allow early intervention through preventative strategies (7).

Studies have shown that the inflammatory response is an important stage in the progression of PPOI (8–10), in which various inflammatory factors are intensively secreted, leading directly or indirectly to the disruption of gastrointestinal motility (11, 12). Significant elevations in cytokines such as IL-6 and TNF-α are often taken as strong evidence for the development of PPOI (13). However, there is still a lack of clear and specific criteria to clarify the relationship between them in clinical settings.

Based on the above, our present study intended to establish a predictive model for PPOI after radical gastrectomy by assessing the inflammatory cytokines. We put our emphasis on postoperative inflammatory mediators in these patients with the expectation of quantifying their relationship with the onset of PPOI, and thus we could clinically identify the high-risk groups for the purpose of early intervention.

## Methods and materials

### Patient section

Our present study selected the inpatients who underwent radical gastrectomy from July 2022 to June 2024 at our center. The inclusion criteria were as follows: (1) 18-80 years old; (2) proposed radical surgery for gastric cancer; (3) signed an informed consent form to voluntarily participate in this trial. The exclusion criteria were as follows: (1) patients with cardiac, hepatic, or renal insufficiency; (2) patients who underwent emergency surgery; (3) other complications such as infection, bleeding, or the necessity for additional operation after the surgery. At last, 222 patients were finalized for inclusion in the study, of which 154 patients from July 2022 to December 2023 served as the training set and the remaining 68 patients were taken as the validation set. All included patients with gastric cancer were operated on by experienced surgeons. This study was approved by the ethics committee of our center and was performed with the informed consent of patients. The entire process followed the ethical standards of Declaration of Helsinki and its later amendments.

### Inflammatory mediators

Through intravenous blood collection on the first postoperative day, we focused on the expression of various inflammatory mediators in the serum of the patients, including IL-2, IL-4, IL-6, IL-10, TNF-α, IFN-γ, and C-reactive protein (CRP). All the above inflammatory mediators were detected by ELISA in our laboratory department.

### Definition of the outcome

The definition of PPOI was referenced to the criteria proposed by recent systematic reviews and global surveys (14), which was adopted by many subsequent studies (5, 15, 16). Specifically, a diagnosis of PPOI is made if the patient meets two or more of the following five criteria on postoperative day 4 or longer: (1) Nausea or vomiting over the preceding 12 h; (2) Inability to tolerate an oral diet over the prior 24 h; (3) Absence of passage of both stool and flatus over the preceding 24 h; (4) Abdominal distention; (5) Radiologic confirmation. The diagnosis of PPOI must be made independently by two experienced surgeons.

### Statistical analysis

All relevant examinations of the collected data were performed by SPSS 25.0 software (IBM Corporation, Armonk, NY, USA) and R software. Continuous variables with non-normally distributed variables were expressed as medians and interquartile ranges (IQR), and were assessed with Mann-Whitney U tests. Categorical data were compared by χ2 test or Fisher’s exact test. Receiver operating characteristic (ROC) curve analysis was used to help determine the cut-off values. Both univariate and multivariate logistic regression analysis were used to identify independent factors for PPOI occurrence. R software (version 4.2.1) was used to construct a nomogram based on multivariate analysis. The area under the ROC curve (AUC) was calculated to assess the prediction accuracy of the model and diagnostic calibration curves were used to assess the goodness-of-fit of the nomogram. Decision Curve Analysis (DCA) was applied to evaluate its clinical utility. P value of less than 0.05 was considered to have statistical significance.

## Results

### Patient characteristics

The clinicopathological characteristics of 154 patients in the training cohort and 68 patients in the validation cohort are presented in **Table 1**. The incidence of PPOI in the two groups were 16.9% and 14.7%, which had similar proportions. In the comparison of other general information, we found that the length of surgery, the expression of IL-10, IFN-γ, and TNF-α were statistically different between the training cohort and the validation cohort. However, age, gender, Body Mass Index (BMI), abdominal surgery history, surgical procedure, the level of IL-2, IL-4, IL-6, and CRP were found no statistical significance.

**Table 1:** Clinicopathological features of enrolled patients.

Characteristics	Training cohort N=154	validation cohort N=68	P value
Age (years)			0.112
<65	86 (55.8%)	30 (44.1%)	
≥65	68 (44.2%)	38 (55.9%)	
Gender			0.527
Male	106 (68.8%)	50 (73.5%)	
Female	48 (31.2%)	18 (26.5%)	
BMI (kg/m2) *	23.20 (21.26, 26.03)	22.92 (20.76,24.83)	0.166
Prior abdominal surgery	40 (26.0%)	16 (23.5%)	0.740
Surgical procedure			0.520
Open surgery	108 (70.1%)	51 (75.0%)	
Laparoscopic surgery	46 (29.9%)	17 (25.0%)	
Extent of resection			0.867
Proximal stomach	10 (6.5%)	4 (5.9%)	
Total stomach	61 (39.6%)	31 (45.6%)	
Distal stomach	81 (52.6%)	32 (47.0%)	
Middle Stomach	2 (1.3%)	1 (1.5%)	
Length of surgery (min) * Inflammatory cytokines *	200 (170, 225)	180 (150,209)	0.004
IL-2 (pg/ml)	1.77 (0.35, 3.33)	2.70 (1.79,3.31)	0.403
IL-4 (pg/ml)	2.17 (1.17, 3.51)	2.71 (1.79,2.71)	0.481
IL-6 (pg/ml)	17.85 (11.04, 27.11)	19.80 (14.26,26.60)	0.247
IL-10 (pg/ml)	5.80 (4.18, 8.27)	7.36 (5.86,10.09)	0.001
IFN-γ (pg/ml)	10.30 (6.86, 19.25)	8.67 (6.77,12.30)	0.012
TNF-α (pg/ml)	2.45 (1.57, 4.28)	3.75 (2.58,4.97)	0.002
CRP (mg/L)	32.15 (18.43, 51.00)	35.60 (22.60,58.00)	0.181
Prolonged postoperative ileus	26 (16.9%)	10 (14.7%)	0.701

*Median (IQR).

### Evaluation of the predictive potential of different inflammatory cytokines

**Figure 1:**
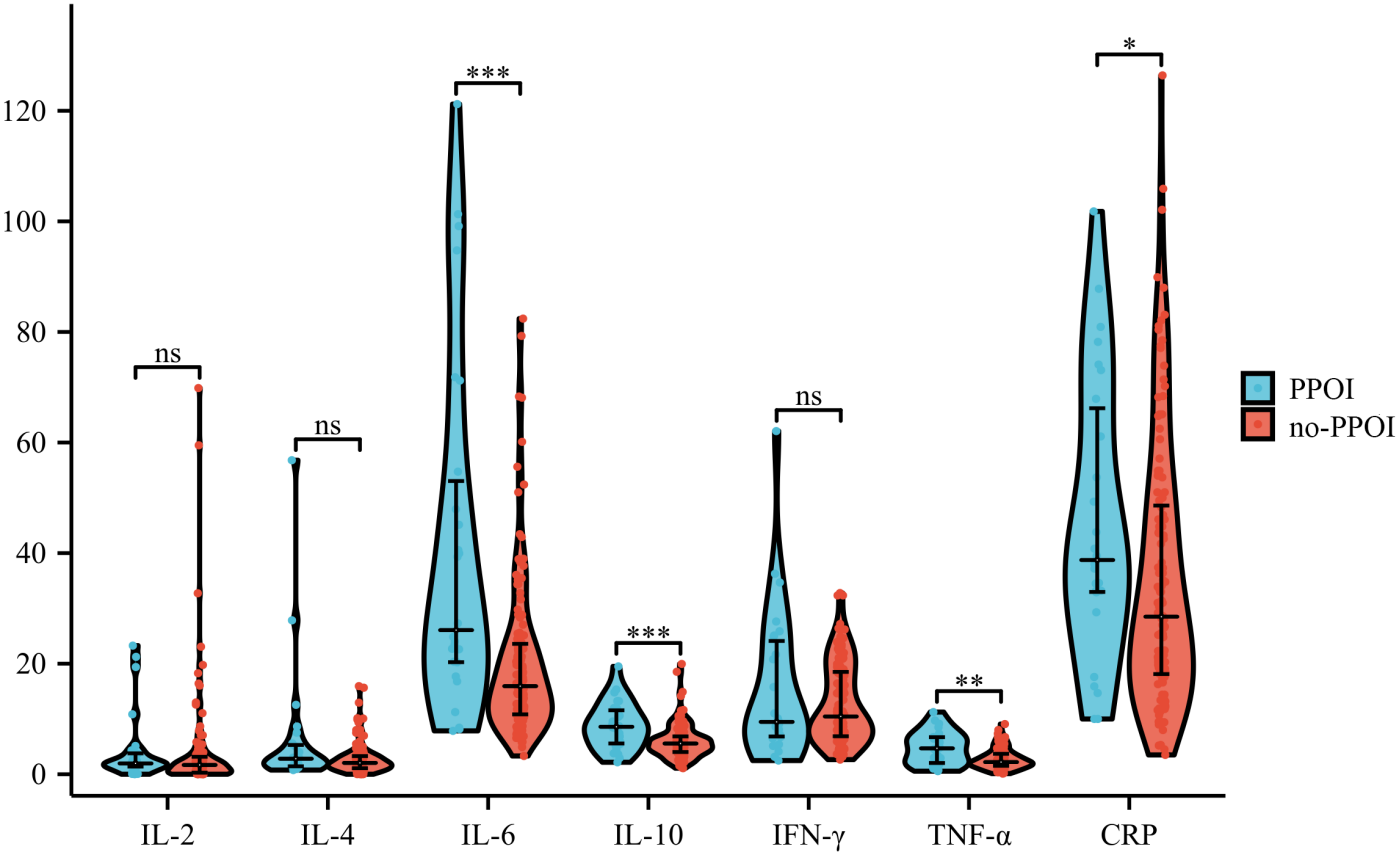
Differences in inflammatory factors of patients with and without PPOI. ViolinPlot shows that IL-6 (P<0.001), IL-10 (P<0.001), TNF-α (P<0.01), and CRP (P<0.05) are upregulated in PPOI patients after surgery, while IL-2, IL-4, and IFN-γ were found no difference.

To initially determine the variations of inflammatory mediators in patients who experienced PPOI, we analyzed the differences between patients who developed and did not develop PPOI in the training group. Results showed that despite a few isolated extremes, the average value of IL-6 (P<0.001), IL-10 (P<0.001), TNF-α (P<0.01), and CRP (P<0.05) in PPOI patients were significantly increased after surgery, while IL-2, IL-4, and IFN-γ were found no difference (shown in **Figure 1**).

**Figure 2:**
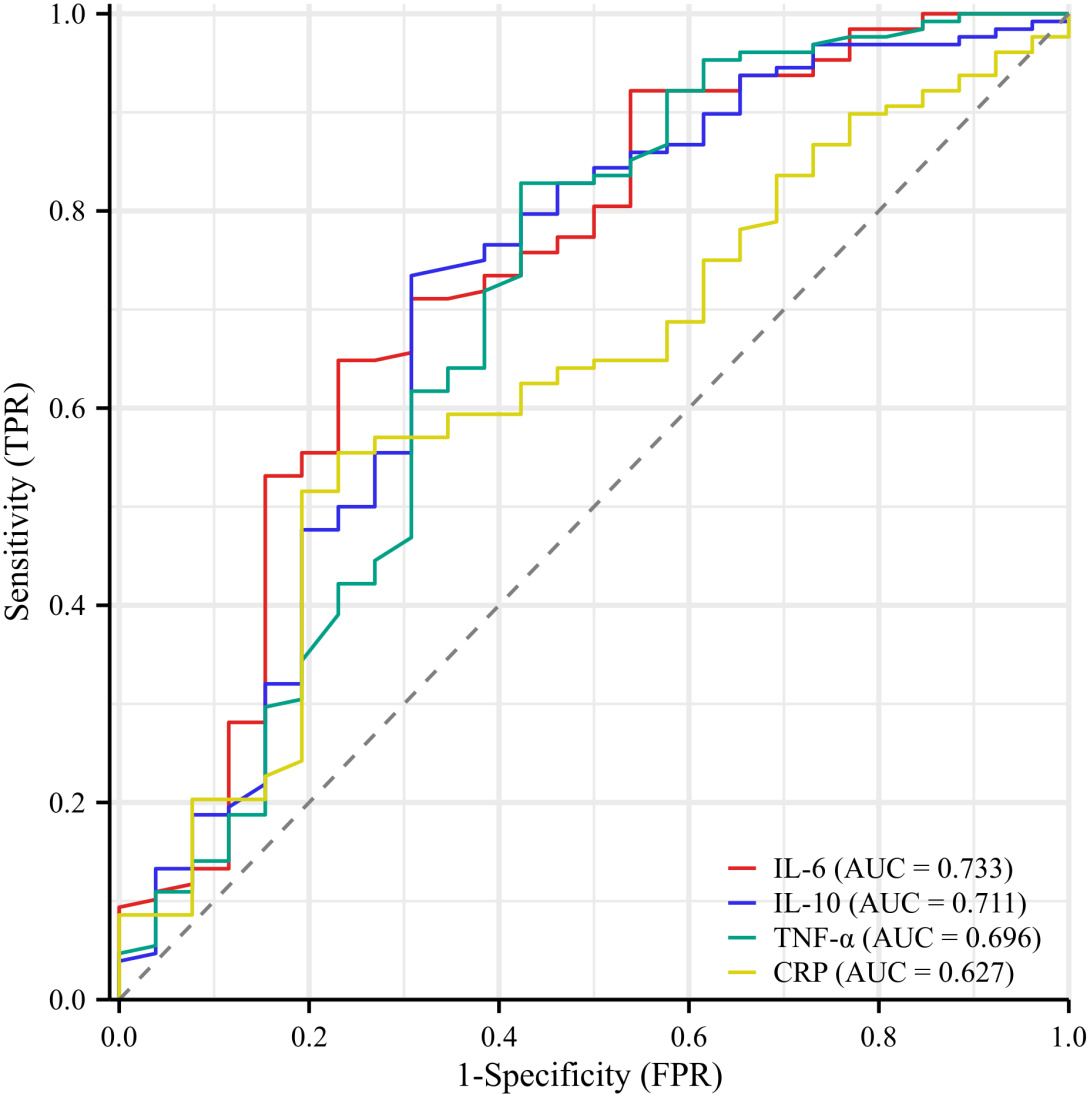
Receiver operator characteristic curve analysis of inflammatory indicators. The AUC were 0.733 for IL-6, 0.711 for IL-10, 0.696 for TNF-α, and 0.627 for CRP.

Subsequently, ROC curve analysis was used to provide a detailed picture of the relationship between the statistically significant indicators mentioned above and the occurrence of PPOI (shown in **Figure 2**). The AUC were 0.733 for IL-6, 0.711 for IL-10, 0.696 for TNF-α, and 0.627 for CRP. By calculating the maximum value of the Youden index, we found the cut-off point was 20.02 for IL-6, 6.78 for IL-10, 4.34 for TNF-α, and 32.15 for CRP. The detailed results were presented in **Table 2**. Based on the cut-off values, we classified these inflammatory mediators into two groups.

**Table 2:** Receiver operator characteristic analyses for IL-6, IL-10, TNF-α and CRP.

	IL-6	IL-10	TNF-α	CRP
AUC (95%CI)	0.733 (0.618~0.849)	0.711 (0.590~0.832)	0.696 (0.568~0.824)	0.627 (0.512~0.742)
Cut-off point	20.02	6.78	4.34	32.15
Sensitivity (%)	76.92	69.23	57.69	76.92
Specificity (%)	64.84	73.44	82.81	55.47
Youden Index	0.418	0.427	0.405	0.324
P value	<0.001	0.001	0.002	0.042

### Univariate and multivariate analysis

Next, we analyzed the risk factors for PPOI occurrence by logistic regression model. Univariate examination showed that age ≥ 65 (P = 0.001, HR = 5.556, 95%CI: 2.085~14.802), IL-6 ≥ 20.0 pg/ml (P <0.001, HR = 6.148, 95%CI: 2.303~16.411), IL-10 ≥ 6.78 pg/ml (P <0.001, HR = 6.221, 95%CI: 2.478~15.618), TNF-α ≥ 4.34 pg/ml (P <0.001, HR = 6.570, 95%CI: 2.662~16.216), and CRP ≥ 32.15 mg/L (P =0.004, HR = 4.152, 95%CI: 1.564~11.026) were significantly related the onset of PPOI. Subsequently, multivariate analysis demonstrated that age ≥ 65 (P =0.002, HR=6.092, 95%CI: 1.946~19.074), IL-6 ≥ 20.0 pg/ml (P = 0.007, HR = 4.639, 95%CI: 1.536~14.011), and IL-10 ≥ 6.78 pg/ml (P = 0.032, HR = 4.044, 95%CI: 1.132~14.455) were predictive factors for PPOI (shown in **Table 3**).

**Table 3:** Logistic regression model analysis for PPOI.

Factors	Univariate analysis		Multivariate analysis	
	OR (95%CI)	P value	OR (95%CI)	P value
Age				
<65	Reference		Reference	
≥65	5.556(2.085~14.802)	**0.001**	6.092(1.946~19.074)	**0.002**
Gender				
Male	Reference			
Female	0.471(0.166~1.334)	0.156		
BMI (kg/m^2^)				
<23.2	Reference			
≥23.2	1.000 (0.430~2.324)	1.000		
Prior abdominal surgery				
No	Reference			
Yes	0.465(0.150~1.443)	0.185		
Surgical procedure				
Open surgery	Reference			
Laparoscopic surgery	1.302(0.533~3.183)	0.563		
Extent of resection				
Total stomach	Reference			
Distal stomach	0.869(0.360~2.099)	0.755		
Others	0.909(0.174~4.746)	0.910		
Length of surgery				
<200min	Reference			
≥200min	1.096(0.471~2.552)	0.832		
IL-6 (pg/ml)				
<20.0	Reference		Reference	
≥20.0	6.148 (2.303~16.411)	**<0.001**	4.639(1.536~14.011)	**0.007**
IL-10 (pg/ml)				
<6.78	Reference		Reference	
≥6.78	6.221(2.478~15.618)	**<0.001**	4.044(1.132~14.455)	**0.032**
TNF-α (pg/ml)				
<4.34	Reference		Reference	
≥4.34	6.570(2.662~16.216)	**<0.001**	2.916(0.848~10.026)	0.089
CRP (mg/L)				
<32.15	Reference		Reference	
≥32.15	4.152(1.564~11.026)	**0.004**	2.607(0.837~8.123)	0.100

The bold values are statistically different.

### Nomogram construction for predicting the likelihood of PPOI onset

**Figure 3:**
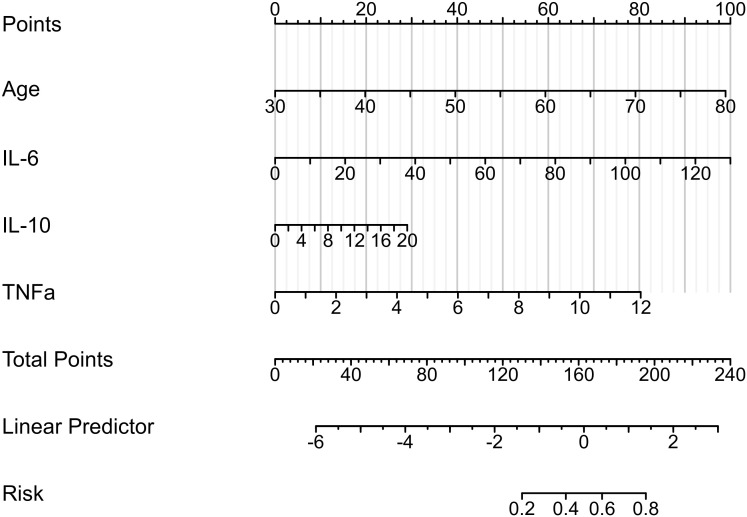
Nomogram to estimate the probability of PPOI. Four variables were finalized: age, IL-6, IL-10, and TNF-α. The linear predictors were calculated as follows: -9.435 + 0.084 × age + 0.033 × IL-6 + 0.062 × IL-10 + 0.284 × TNF-α.

Based on the above-mentioned analysis of the predictive potential for the included clinical variables, we progressively filtered out the variables and established the stepwise nomogram to estimate the probability of PPOI. Meanwhile, ROC curve analysis was applied to measure the performance of the model. At last, four variables were finalized: age, IL-6, IL-10, and TNF-α. The ultimate nomogram was shown in **Figure 3** and the linear predictors were calculated as follows: -9.435 + 0.084 **×** age **+** 0.033 **×** IL-6 + 0.062 **×** IL-10 + 0.284 **×** TNF-α.

### Validation of the model

**Figure 4:**
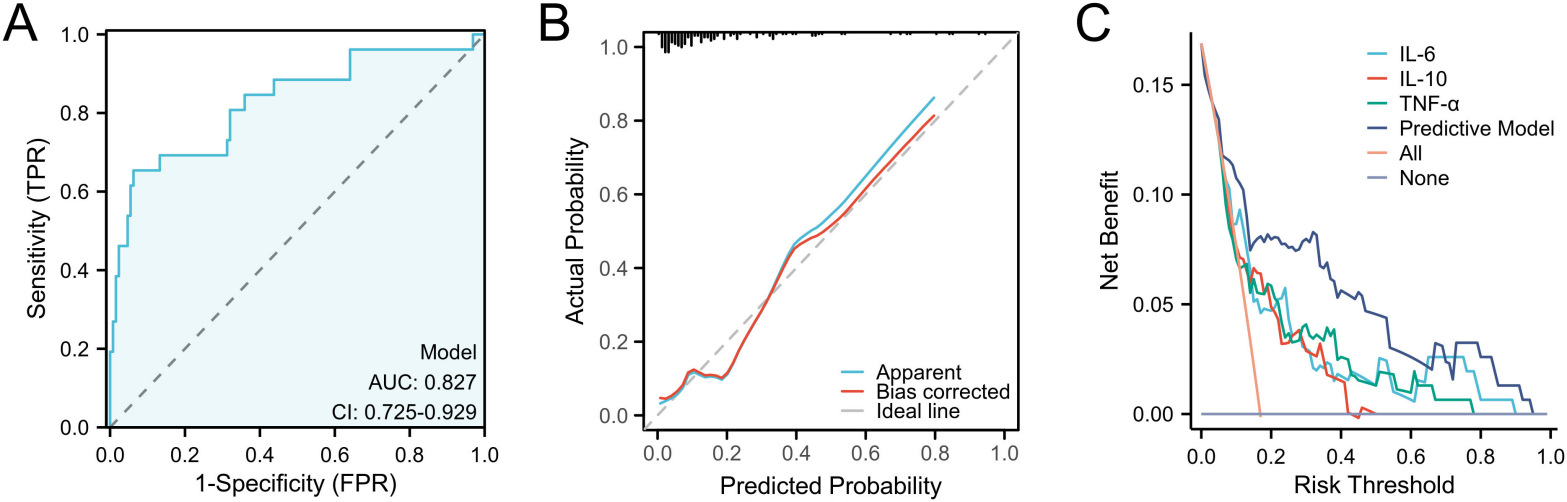
Predictive ability evaluation of the nomogram by training set. **(A)** ROC curve analysis. **(B)** internal bootstrap validation calibration curve. **(C)** decision curve analysis.

**Figure 5:**
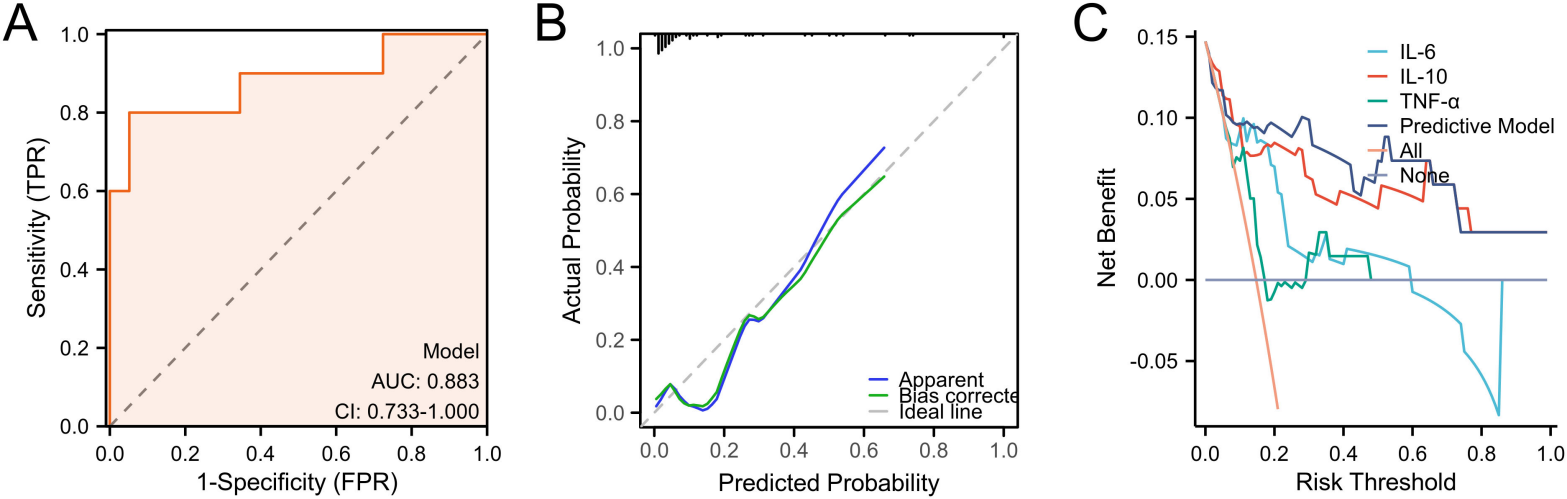
Predictive ability evaluation of the nomogram by validation set. **(A)** ROC curve analysis. **(B)** internal bootstrap validation calibration curve. **(C)** decision curve analysis.

After completing the modeling, we further evaluated the predictive ability of the nomogram. We first validated the training set by ROC curve analysis. The AUC of the model was 0.827 (95%CI: 0.725-0.929), suggesting a relatively good diagnostic potential (**Figure 4A**). The internal bootstrap validation calibration curve showed a relatively good fit compared to the ideal curve after 200 times sampling (**Figure 4B**). Furthermore, decision curve analysis (DCA) showed that the model generally had good positive net benefits (**Figure 4C**). Subsequently, we further analyzed the predictive efficacy of the variables included in the model through the validation set (**Figure 5**). The AUC reached 0.883 with the inclusion of the same variables. The validation calibration curve and the DCA curve of the validation set showed a similar result. The model may overestimate the incidence of PPOI when the probability becomes larger. Nevertheless, it still presented a potentially favorable clinical benefit.

## Discussion

PPOI is a relatively common postoperative complication following the abdominal surgery (17). Particularly in patients with gastric cancer, extensive intraoperative manipulations often result in disruption of the intestinal transit function, leaving gut rehabilitation as a clinical priority (18, 19). To avoid further suffering from this disorder, earlier prediction of exposure to PPOI is therefore urgently required (20, 21). Currently, an immunological and inflammatory response which begins 3-4h after surgery and lasts for several days has been demonstrated to play a crucial role in the prolonged duration of PPOI (22, 23). During this phase, a large number of immune cells were recruited or activated and subsequently secreted numerous inflammatory factors such as IL-6, IL-10, TNF-α and IFN-γ (24–26). These inflammatory mediators have been shown in extensively animal experiments to affect intestinal motility through a variety of means and have been used as potential indicators of PPOI occurrence (27, 28). Despite sufficient evidence in the animal models, there have been few studies on these inflammatory mediators in clinical PPOI patients, and there are no definitive reports on their association with the incidence of PPOI.

Therefore, our present study focused on discussing which specific inflammatory mediators were indicative of PPOI after gastric cancer surgery. Our results revealed that the expression of IL-6, IL-10, TNF-α, and CRP was elevated in PPOI patients. Through subsequent univariate and multivariate analysis, IL-6, IL-10 and TNF-α were demonstrated to be strongly related to the PPOI occurrence, which was consistent with numerous basic research. It can be inferred that during the development of PPOI in these patients, massive resident inflammatory cells are activated while numerous immune cells are recruited, subsequently resulting in the secretion of inflammatory mediators. Consequently, our studies of inflammatory factors in clinical patients such as IL-6, IL-10 and TNF-α provided further complementary evidence on the development of the inflammatory phase of PPOI. In addition, our study demonstrated that advanced age was an independent risk factor for PPOI onset, in line with several previous findings. Due to the limitations of a retrospective study, all ELISA assay data were derived from clinical records generated during patient consultations. According to the standard operating procedures of the hospital laboratory, the number of assays was determined by actual clinical practice, and no additional technical duplications were added. Typically, elderly patients have prolonged postoperative recovery time and increased disruption of intestinal motility caused by surgical trauma. However, clinical characteristics such as prior abdominal surgery, surgical procedure and length of surgery were found no correlation to PPOI in our study. More clinical samples may be necessary to analyze the exact relationship of these factors to PPOI in our center.

Studies of the occurrence of PPOI could play an essential role in the development of new strategies to prevent or reduce this disorder. Of all the 222 patients with gastric cancer enrolled in our present study, 36 developed PPOI, accounting for 16.2%. We developed a nomogram to calculate the exact probability of PPOI in patients underwent radical gastrectomy. This is especially beneficial for physicians in assessing risk in advance, making timely clinical decisions, and communicating with patients when appropriate. Our prediction model primarily incorporated the postoperative inflammatory mediators as independent variables. The good predictive accuracy and application value of this model were demonstrated by the confirmation of the training and validation sets. To our knowledge, this is the first inflammatory factor-based prediction model for PPOI in gastric cancer patients, and it provides early warning signals of PPOI in gastric cancer patients from a new perspective, allowing more selectivity for early clinical detection and timely intervention. For patients at higher risk of PPOI, strategies such as optimizing fluid management, reducing opioid use, encouraging early activities out of bed, and promoting gum chewing should be maximized in the postoperative period. In addition, special attention should be paid to postoperative monitoring in these patients to prevent pulmonary infection, deep vein thrombosis, and other possible complications after prolonged hospital confinement. At the same time, our study had several limitations. Firstly, the exact cut-off time regarding the persistence of bowel obstruction is not widely accepted due to the different type of abdominal surgery. it is necessary to have a standardized and universally accepted definition of the exact point at which POI changes into PPOI. Secondly, since this was a single-center study, the lack of strong external validation and the relatively small amount of data may have weakened the results of the analysis. Thus, follow-up studies are needed to further complement and confirm the existing results.

## Conclusion

We developed a novel prediction nomogram based on the level of inflammatory mediators on the first operative day for gastric cancer. This model may help us clinically identify the high-risk groups of PPOI for the purpose of early intervention.

## Data Availability

The raw data supporting the conclusions of this article will be made available by the authors, without undue reservation.
